# Exploring the Use of Smartwatches and Activity Trackers for Health-Related Purposes for Children Aged 5 to 11 years: Systematic Review

**DOI:** 10.2196/62944

**Published:** 2025-01-27

**Authors:** Lauren Thompson, Sydney Charitos, Jon Bird, Paul Marshall, Amberly Brigden

**Affiliations:** 1 University of Bristol Bristol United Kingdom

**Keywords:** children, systematic review, wearable activity trackers, smartwatches, feasibility, mobile phone

## Abstract

**Background:**

Digital health interventions targeting behavior change are promising in adults and adolescents; however, less attention has been given to younger children. The proliferation of wearables, such as smartwatches and activity trackers, that support the collection of and reflection on personal health data highlights an opportunity to consider novel approaches to supporting health in young children (aged 5-11 y).

**Objective:**

This review aims to investigate how smartwatches and activity trackers have been used across child health interventions (for children aged 5-11 y) for different health areas, specifically to identify the population characteristics of those being targeted, describe the characteristics of the devices being used, and report the feasibility and acceptability of these devices for health-related applications with children.

**Methods:**

We searched 10 databases (CINAHL, Embase, ACM Digital Library, IEEE Xplore, Cochrane Library, PsycINFO, Web of Science, PubMed, Scopus, and MEDLINE) to identify relevant literature in March 2023. The inclusion criteria for studies were as follows: (1) peer-reviewed, empirical studies; (2) published in English; (3) involved a child aged 5 to 11 years using a smartwatch for health-related purposes. Two researchers independently screened articles to assess eligibility. One researcher extracted data relating to the 3 aims and synthesized the results using narrative and thematic synthesis.

**Results:**

The database searches identified 3312 articles, of which 15 (0.45%) were included in this review. Three (20%) articles referred to the same intervention. In 77% (10/13) of the studies, the devices were used to target improvements in physical activity. Other applications included using smartwatches to deliver interventions for emotional regulation and asthma management. In total, 9 commercial devices were identified, many of which delivered minimal data feedback on the smartwatch or activity tracker, instead relying on a partner app running on a linked parental smartphone with greater functionality. Of the 13 studies, 8 (62%) used devices designed for adults rather than children. User feedback was positive overall, demonstrating the acceptability and feasibility of using these devices with children. However, the studies often lacked a child-focused approach, with 3 (23%) studies gathering user feedback only from parents.

**Conclusions:**

Interventions involving smartwatches and activity trackers for children aged 5 to 11 years remain limited, primarily focusing on enhancing physical activity, with few studies investigating other health applications. These devices often provide limited data feedback and functionality to support children’s independent engagement with the data, relying on paired smartphone apps managed by caregivers, who control access and facilitate children’s interaction with the data. Future research should adopt child-centered methods in the design and evaluation of these technologies, integrating children’s perspectives alongside their caregivers, to ensure that they are not only feasible and acceptable but also meaningful and effective for young children.

**Trial Registration:**

PROSPERO CRD42022373813, https://tinyurl.com/4kxu8zss

## Introduction

### Background

Health during childhood is a key predictor of health outcomes in adolescence and adulthood [1-5]. Poor physical health during childhood that continues into adulthood can result in higher health care costs and poorer quality of life [6]. As the prevalence of childhood ill health increases [7], it is important to consider novel approaches to help improve health outcomes in children. Behavioral interventions can be used to promote positive lifestyle behaviors (eg, engaging in physical activity [PA]) as well as support the management of chronic conditions (eg, promoting treatment adherence) [8].

For adults and adolescents, there is evidence that digital health interventions are effective in promoting health behavior change while also being cost-effective, appealing, and accessible [9-11]. However, in a systematic review of digital health interventions for children, of the 17 digital interventions identified, only 5 were found to be promising in producing a positive impact on clinical outcomes [12]. The lack of effective interventions indicates a need for progress in the field of digital health interventions for children, investigating technological innovations that meet the specific needs and contexts of children.

The development of wearables and sensors presents innovative solutions for delivering behavioral interventions. There are now a large number of commercial products that enable the real-time tracking and monitoring of personal health information and behaviors such as step count, heart rate, and sleep [13]. Devices such as smartwatches are well-suited for use as health intervention tools, as they are widely available and allow users to reflect on the health information collected via sensors in real time to gain a better understanding of their health and engage in self-management behaviors [14]. In this review, we define a smartwatch as a wrist-worn device that uses sensors to collect at least 2 types of personal health data (eg, steps and heart rate) and has an interactive display. With the popularity of adult trackers, there is also an established market for wearables and smartwatches for children [15]. These devices may be more appropriate for younger users, as they can be used independently from mobile devices, as device ownership is uncommon in young children [16].

There is a growing body of evidence for activity tracker and smartwatch health interventions for adults and adolescents, but despite the promise, there has been inadequate attention to their use with children [17,18]. Designing and deploying wearable health interventions for children needs specific attention due to the distinct developmental characteristics of this age group (social, emotional, and cognitive) and their greater reliance on parents and carers for support [8,19,20]. This includes considering how wearable hardware and software should be designed to meet the distinct needs of children. It also includes considering wider systems, such as family systems, for example, considering how multiple devices should be integrated into one intervention to meet the needs of different users (eg, adult and child) and how issues such as wearable data sharing and data privacy should be navigated. To understand these technical and sociotechnical issues, understanding the end users’ experience, such as the technology’s feasibility and acceptability, is essential [21].

Few reviews have focused on the design and user experience of wearables for children. Systematic reviews of the acceptability and feasibility of wearable devices to promote PA in children have identified that wearables can increase children’s motivation for PA using behavior change techniques but noted novelty effects and uncertainties over long-term use and impact [17,18]. However, these reviews had a narrow focus on wearables for PA and a broad age range; they were not designed to explore developmentally sensitive design implications for children across health contexts. Previous work has identified common design adaptations for children’s devices such as being more colorful, having fewer health metrics (eg, calories burned not included), and including features, such as games, to boost children’s engagement [15,22]. However, these did not use a systematic review methodology and are limited to presenting a description of design adaptations without analysis of the end users’ experiences or engagement with these design adaptations.

### Aims

This systematic review aimed to investigate how smartwatches and activity trackers have been used across the full range of child health interventions (for children aged 5-11 y), spanning public health and prevention interventions to interventions for clinical populations, focusing on their acceptability and feasibility for children. This review aimed to build on the established literature on digital interventions for children by investigating design considerations to support young children’s engagement with the smartwatch and the data collected for their health. Therefore, we focused on devices with an interactive display that supports data feedback. In this review, we investigated the following research questions (RQs): (1) What are the population characteristics of those being targeted in the health-related smartwatch and activity tracker interventions for children aged 5 to 11 years? (2) What are the characteristics of the smartwatches and activity trackers being used for health-related applications with children aged 5 to 11 years (eg, device type and features included)? (3) What is the feasibility and acceptability of using smartwatches and activity trackers for health-related applications with children aged 5 to 11 years?

## Methods

This review was registered in the PROSPERO database and follows the guidance of the PRISMA (Preferred Reporting Items for Systematic Reviews and Meta-Analyses) guidelines [23]. See Multimedia Appendix 1 for the full checklist.

### Search Strategy

The literature search was conducted in March 2023 in the following databases: CINAHL, ACM Digital Library, PsycINFO, Embase, MEDLINE, Cochrane Library, IEEE Xplore, PubMed, Scopus, and Web of Science. The search included key terms for (1) children aged 5 to 11 years, such as “school child” or “minor” and (2) smartwatches, such as “smartwatch” or “wearable electronic device” (Multimedia Appendix 2 provides an example of the full list of search terms used in Embase).

### Inclusion and Exclusion Criteria

Studies were included if they fulfilled the following criteria: (1) peer-reviewed or empirical studies with any study design; (2) published in English; and (3) the research involved a child (aged 5-11 y) using a smartwatch for health-related purpose, including behavior change intervention (physical, mental, or social). We use the term intervention to apply to any activity undertaken to improve health [24]. For the purpose of this review, a smartwatch is defined as a wrist-worn device that uses sensors to collect at least 2 types of personal health data (eg, steps and heart rate) and has an interactive display. We also extended this to include activity trackers with a visual display (excluding those that do not reflect on the data via the device), as they are largely similar to smartwatches but are typically cheaper with fewer sensors and can be used without a smartphone [25]. We selected devices that collect at least 2 types of personal health information and incorporate a visual, interactive display, thereby excluding simpler devices such as pedometers, which lack these functionalities and therefore do not meet our criteria of being a smartwatch or activity tracker. The devices could be used in conjunction with a partnering app if the child engages with the wearable device independently as part of its use.

Studies were excluded if they fulfilled the following criteria: (1) the study did not use a smartwatch or activity tracker with an interactive display; (2) the children in the study only wore the smartwatch or activity tracker as a passive data collection tool, and they did not otherwise interact with the device, by which we mean actively collect or view the data it collected as part of an intervention or feasibility assessment (eg, accuracy or validation studies); (3) the study included children aged <5 years or >11 years or do not report on the eligible age range specifically; or (4) the article was considered a review on previously reported studies, protocol, nonempirical or nonscientific paper (eg, book chapters), or when the full text was not available.

### Screening Procedures

The search results were imported into Zotero (Corporation for Digital Scholarship) and duplicates were removed. Each title and abstract were independently screened by 2 authors (LT and SC) for relevance. If an inclusion decision could not be made based on the title and abstract, the articles were included for full-text review. Articles deemed relevant at the title and abstract screening stage were independently double-screened against the inclusion and exclusion criteria using the data management platform Rayaan (Rayyan Systems Inc), with reasons for exclusion recorded. Disagreements at both stages were discussed and resolved in meetings by the reviewers (LT and SC), with persistent disagreements being resolved through discussion with the wider research team (AB and JB). If the full text did not contain the information needed, 2 attempts were made to contact authors by email; if the information was not provided, the study was excluded.

### Data Extraction and Synthesis

Data related to our RQs were extracted by the primary author (LT), as described in Textbox 1. Due to the heterogeneity in the study designs and the data reported, we synthesized data using narrative and thematic synthesis [26,27]. The extracted data are summarized in Textbox 1. For qualitative data, open coding was conducted by the primary author on the available qualitative data referring to participants’ experiences of using the device, which included quotations from participants or authors’ interpretations of participants’ experiences. Themes were developed top-down using the technology acceptance model (TAM) [28], in which codes generated were grouped under each relevant theme of the TAM (perceived ease of use and perceived usefulness). The remaining analysis was data driven (bottom-up) to develop themes for any codes that fell outside the scope of the TAM. Wherever possible, quotes from the participants of the included studies (primary data) have been presented. In addition, to understand the characteristics of the device relevant to behavior change, we used the taxonomy of app features [29] and coded whether the features were present on the smartwatch device or partner app in each research study as shown in Multimedia Appendix 3.

**Data extraction categories and details extracted.**

**General study information**

- Publication details: authors, year of publication, country of study, and publication location
- Study aim
- Sample size

**Research question 1: What are the population characteristics of those being targeted in the health-related smartwatch or activity tracker interventions?**

- Health behavior or condition targeted
- Stakeholders involved (eg, target child, parent, or other)
- Child sample characteristics: age and gender
- Child’s device experience

**Research question 2: What are the characteristics of the smartwatch or activity trackers?**

- Intervention summary: a description of the wearable element of the intervention
- Device details: name, type (smartwatch or activity tracker), technology readiness level [30] (consumer device or experimental prototype), and if there is a partner app or device
- Device rationale and justification: information that the authors provide describing why the device was selected
- Smartwatch software details: description of features, functionality, and interaction modality and whether the software used was modified or bespoke, including any justification or rationale for design choices
- Software on partner app: using the taxonomy of app features [29] to identify app features used to promote behavior change
- Data sharing features: who the data is shared with and if data sharing is part of the interventions

**Research question 3: What is the feasibility and acceptability of using a smartwatch and activity tracker for health-related applications with children aged 5 to 11 years?**

- Qualitative data relating to acceptability and feasibility and who reported these data (eg, child or parent)
- Quantitative data relating to acceptability and feasibility (eg, measures of use and who reported these data)
- Any information captured about acceptability and feasibility but not captured through formal qualitative or quantitative data collection, that is, authors’ interpretations in the results and discussion

### Quality Assessment

Two authors conducted quality assessments independently, discussing disagreements to reach a consensus. As the studies varied in study design, we used different quality assessment tools. We used the Critical Appraisal Skills Programme systematic review checklist [31] for qualitative studies. This is a 10-item checklist with 3 responses: “yes,” “no,” and “cannot tell.” The Quality of Survey Studies in Psychology checklist [32] was used to assess articles that collected user feedback via surveys and questionnaires. This checklist comprises 20 items under 4 categories: introduction, participants, data, and ethics. Finally, the National Heart, Lung, and Blood Institute pre-post and controlled studies assessment tools were used. These are 12- and 14-item checklists with 5 responses: “yes,” “no,” “cannot determine,” “not applicable,” and “not reported” [33,34]. Full details of the quality assessment can be found in Multimedia Appendix 4 [35-49].

## Results

In this section, we summarize the data extracted to address our RQs as outlined in Textbox 1.

### Search Results

**Figure 1:**
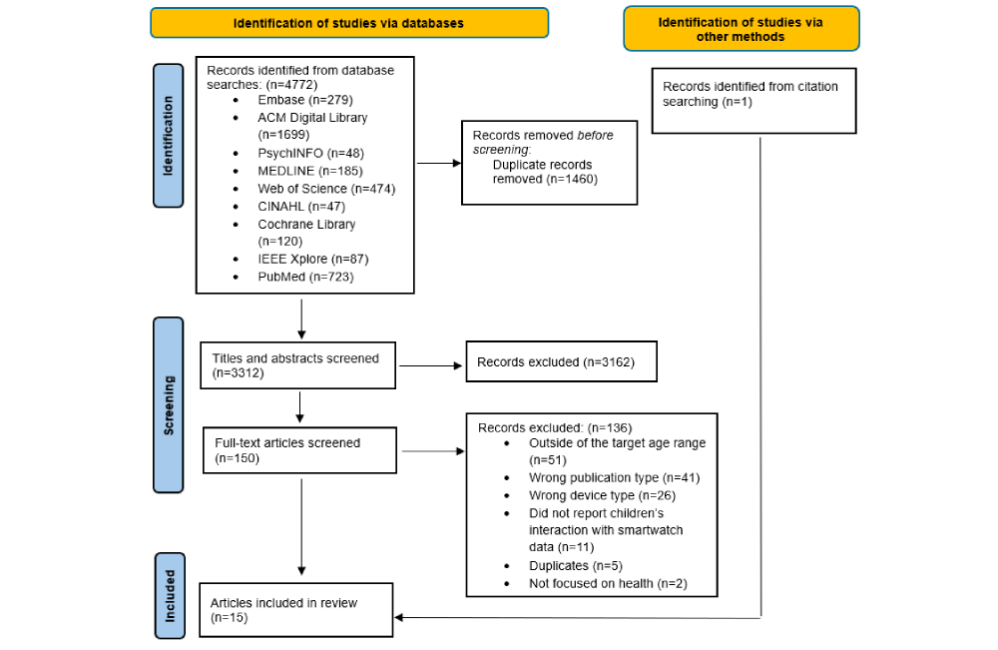
PRISMA (Preferred Reporting Items for Systematic Reviews and Meta-Analyses) flow diagram.

After deduplication, 3312 (69%) of the 4772 articles were identified from the database searches. After abstract and title screening, 150 (4.5%) articles were considered for a full-text review, and of these, 15 (10%) were identified as eligible for inclusion. In total, 3 articles reported on the same intervention; therefore, we identified 13 studies detailing children’s (aged 5-11 y) use of smartwatches for health-related purposes. A summary of the study details can be found in Table 1. Table 2 provides a summary of the population characteristics. We will refer to these 13 distinct studies unless otherwise indicated. Most (11/13, 85%) studies focused on using a single device, but 2 (15%) studies [35,36] used multiple devices during the study period. Additional devices not meeting the study criteria (n=5) are not reported here. The most common reasons for exclusion were participants being outside the target age range, wrong publication type, and wrong device type. The PRISMA flow diagram can be found in Figure 1.

**Table 1:** Summary of included studies.

Study	Location	Sample size	Summary
O’Brien et al [37], 2020	NR^a^	1 child	Investigated the acceptability of a smartwatch intervention that sends visual directives to children with autism spectrum disorder
Buchele Harris and Chen [38], 2018	NR	116 children	Investigated the impact of a technology-enhanced physical activity classroom intervention
Saksono et al [39], 2018	United States	9 children and 11 parents	Explored the experiences of low SES^b^ families using physical activity tracking technologies
Schoeppe et al [40], 2020	Australia	40 families; 58 children	Examined the short-term effects of an activity tracker and app intervention to increase physical activity in families (Step it Up Family program)
Schoeppe et al [41], 2023	Australia	19 families	Explored families’ experience and satisfaction with a physical activity intervention using wearable activity trackers and apps (Step it Up Family program)
Schoeppe et al [42], 2022	Australia	40 families; 58 children	Explored the feasibility of a family-based activity tracker intervention to increase physical activity (Step it Up Family program)
Torrado et al [43], 2017	Spain	2 children	Assessed the usability of a personalized emotional self-regulation tool
Creaser et al [44], 2022	United Kingdom	36 adults and 29 target children	A mixed methods observational study that explored the acceptability of using wearables in a family setting
Masteller et al [36], 2017	NR	16 children	Explored children’s perceptions of activity trackers and their associated websites
Wing et al [45], 2022	United States	137 children	Explored the usability and acceptability of Fitbit Charge with families via pre- and poststudy questionnaires
Hosseini et al [46], 2017	United States	1 adult and 1 child	Pilot study to assess the feasibility of the mobile health asthma system in a real-world setting
Jackson et al [47], 2022^c^	United States	64 children; 35 children; 39 children; 56 children; 38 children; 25 children	Evaluated the impact of Fitbit Charge HR on children’s activity levels during school
Schaefer et al [35], 2014	United States	25 children	A qualitative study to assess the acceptability and compliance of wearable activity trackers
Duck et al [48], 2021	United States	35 children	A quasi-experiment to assess the effectiveness of an activity tracker with altruistic motivation at increasing physical activity behavior
Saksono et al [49], 2019	United States	16 parents and 15 children	A qualitative study investigating how parents and children interact with their activity trackers in a naturalistic low SES context

^a^NR: not reported.

^b^SES: socioeconomic status.

^c^This study reported several conditions with unique participant demographics and study procedures.

**Table 2:** Summary of population characteristics (research question 1).

Study	Health focus	Age range (y)	Motivation for demographics?	Stakeholders involved in the intervention	Setting	Device experience
O’Brien et al [37], 2020	Autism spectrum disorder	9	No	Teacher	School	—^a^
Buchele Harris and Chen [38], 2018	Physical activity	10-11	No	Teacher	School	—
Saksono et al [39], 2018	Physical activity	5-11	No	Parents	Family intervention	—
Schoeppe et al [40], 2020; Schoeppe et al [41], 2023; Schoeppe et al [42], 2022	Physical activity	6-10	Yes; children aged 6 to 10 years were targeted as this age range is important for forming physical activity behaviors	Parents and siblings	Family intervention	Required to have none for eligibility
Torrado et al [43], 2017	Autism spectrum disorder	10	No	Parents and teachers	School	Yes
Creaser et al [44], 2022	Physical activity	5-9	No	Parents and siblings	Family intervention	Yes; 4 children owned a device; duration of use ranged from <1 month and >2 years
Masteller et al [36], 2017	Physical activity	6-11	Yes; elementary school children were the target demographic for the wearable	Parent	—	—
Wing et al [45], 2022	Physical activity	9-10	No	Parents	—	—
Hosseini et al [46], 2017	Asthma	7	No	—	—	—
Jackson et al [47], 2022	Physical activity	9-10	No	—	School	—
Schaefer et al [35], 2014	Physical activity	7-10	No	Parents	—	—
Duck et al [48], 2021	Physical activity	9-10	No	—	School	—
Saksono et al [49], 2018	Physical activity	6-11	No	Parents	—	—

^a^Not applicable.

### RQ1: What Are the Population Characteristics of Those Being Targeted in Health-Related Smartwatch and Activity Tracker Interventions?

#### Health Behaviors Targeted

Most studies (10/13, 77%) targeted PA behavior. The remaining 23% (3/13) of the studies targeted specific health conditions or recruited participants from specific demographic groups, including autism spectrum disorder (ASD; n=2, 15%) and asthma (n=1, 8%).

#### Demographics of Child Participants

Of the 13 studies with >1 participant, where information was provided, the mean child age ranged from 6 to 10 years, and all but one reported that at least 50% of the sample was female. Only 2 (15%) of the studies reported their motivation for the selected age range of participants. Reasons included (1) targeting elementary school-aged children as they were the target demographic of the wearable being used [36] and (2) focusing on children aged 6 to 10 years due to the developmental importance of forming PA behaviors, parental social support, and role modeling. The authors also reported that children aged <5 years were excluded due to concerns about their ability to comprehend and engage with the smartwatch and partner app [40,42].

#### Stakeholders Involved in the Intervention

In total, 3 of the interventions were family-based PA interventions [39-42,44], one of which extended beyond immediate family (parents and siblings) to include stepparents, cousins, and grandparents [44].

Of the 13 studies, 5 (38%) took place within a school setting in which teachers played a role in data collection or had a facilitating role in the intervention [37,38,43,47,48].

#### Device Experience

In total, 2 (15%) of the 13 studies reported on children’s previous experience using smartwatches or activity trackers. In one study, 86.2% of the target group had never owned one. Only 4 children currently owned one, and the duration of use ranged from <1 month to >2 years [44]. In one study [43], both children had previous experience wearing a smartwatch so neither they nor their classroom peers found them distracting. In contrast, child participants in the Step It Up Family intervention [40-42] had to have no previous experience using an activity tracker to be eligible to take part in the study.

### RQ2: What Are the Characteristics of Smartwatch and Activity Trackers Used for Health-Related Applications With Children Aged 5 to 11 Years?

#### Device Details

A total of 9 different devices were used. All of the studies used commercial devices, with Fitbit, Garmin, and UNICEF Kid Power Band being the most common. Three of the selected devices (UNICEF Kid Power Band, Garmin Vivofit Jr, and Movband) are specifically designed for children. Seven devices were described as having a partner app. A summary of the devices and the features reported can be found in Tables 3 and 4.

**Table 3:** Summary of device characteristics (research question 2)^a^.

Study	Device	Bespoke software design?	Partner app; partner app owner	Motivation for device choice or design considerations reported?	Duration of use	Wearable part of a wider intervention?
O’Brien et al [37], 2020	Apple Watch	No	No	Yes; SMS text messages were used to deliver intervention via smartwatch rather than images due to the speed of transfer compared to images	16 days	No
Buchele Harris and Chen [38], 2018	Fitbit Charge HR	No	No	No	5 school days, for 4 weeks	Yes
Saksono et al [39], 2018	UNICEF Kid Power Band	No	Yes; not reported	Yes; accuracy, ease of use, comfort, on-band display and battery life, tailored for children	2 months	No
Schoeppe et al [40], 2020; Schoeppe et al [41], 2023; Schoeppe et al [42], 2022	Garmin Vivofit Jr	No	Yes; parent’s phone	Yes; decision based on feasibility demonstrated previously	6 weeks	Yes
Torrado et al [43], 2017	LG Watch Urbane	Yes	Yes; parent’s phone	Yes; they used recognized pictograms that children had familiarity with	4 hours a day, for 9 days (total of 36 h wear)	No
Creaser et al [44], 2022	Fitbit Alta HR	No	Yes; not reported	No	4 weeks	No
Masteller et al [36], 2017	Movband	No	Yes; website	No	4 days	No
Wing et al [45], 2022	Fitbit Charge HR	No	Yes; parent’s phone or child’s if they had their own	No	22 days	No
Hosseini et al [46], 2017	Samsung Gear Live	Yes	Yes; parent’s phone	Yes; an animated dragon was considered to be more engaging for children	Several weeks	Yes
Jackson et al [47], 2022	Fitbit Charge HR	No	No	Yes; previous research had determined it appropriate to use with children	8 days to 8 weeks	No
Schaefer et al [35], 2014	Polar Active	No	No	No	5 days	No
Duck et al [48], 2021	UNICEF Kid Power Band	No	Yes; child’s tablet	Yes; the device reward system used the theory of altruistic motivation	10 weeks	Yes
Saksono et al [49], 2019	UNICEF Kid Power Band	No	Yes; not reported	Yes; accuracy, ease of use, comfort, on-band display and battery life, tailored for children	2 months	No

^a^In this paper “bespoke software” design refers to any feature that is not present in existing commercial devices.

**Table 4:** Summary of the wearable devices used (research question 2).

Study	Device	Device type	Target user	Interaction modality	Measures on display	Features in the partner app
Saksono et al [39], 2018; Duck et al [48], 2021; Saksono et al [49], 2019	UNICEF Kid Power Band	Activity tracker	Children	Not reported	Not reported	Reward points
Creaser et al [44], 2022	Fitbit Alta HR	Activity tracker	Adults	Touch (tap display and vibration)	Daily steps, distance traveled, calories burned, active minutes, and heart rate	Goal setting, reminders, challenges, rewards, information sharing, and tracking of further health behaviors (eg, sleep)
Torrado et al [43], 2017	LG Watch Urbane	Smartwatch	Adults	Touch (tap display and vibration), auditory	Personalized self-regulation strategy (eg, pictogram of listening to music)	—^a^
O’Brien et al [37], 2020	Apple Watch	Smartwatch	Adults	Touch (vibration), auditory	Not reported	Not reported
Schoeppe et al [40], 2020; Schoeppe et al [41], 2023; Schoeppe et al [42], 2022	Garmin Vivofit Jr	Activity tracker	Children	Touch (vibration)	Daily steps, goal progress (60 min of activity)	Family leaderboard, challenges, goal setting, personal name, animal images on display, rewards (virtual coins), and virtual interactive game
Hosseini et al [46], 2017	Samsung Gear Live	Smartwatch	Adults	Touch (tap display)	Asthma attack risk (happy, neutral, or sad dragon)	Trend lines for each sensor and risk level, enabling users to focus on points of interest and review data over the last several hours. The app also had a study coordinator and physician interface
Masteller et al [36], 2017	Movband	Activity tracker	Children	Not reported	Daily steps (moves)	Displays collected points, steps, miles, and activity averages
Schaefer et al [35], 2014	Polar Active	Smartwatch	Adults	Not reported	Daily activity bar (amount of time in moderate-to-vigorous intensity zones) and animated figure that indicated activity intensity	—
Buchele Harris and Chen [38], 2018; Wing et al [45],2022; Jackson et al [47], 2022	Fitbit Charge HR	Activity tracker	Adults	Not reported	Daily steps, distance traveled, calories burned, and heart rate	Not reported

^a^Not applicable.

#### Device Rationale and Justification

Of the 13 studies, 5 (38%) reported their motivation for the wearable chosen for the study. The Garmin Vivofit Jr was selected due to the author’s review of previous research, which demonstrated high feasibility for monitoring PA in children [40]. Similarly, the Fitbit Charge HR was selected as it had previously been considered appropriate for children [47]. The UNICEF Kid Power wristbands were selected in 2 (15%) studies due to their accuracy, battery life, comfort, on-band display, and age-appropriate design [39,49]. This device was also used in an additional study that investigated the impact of altruistic motivation due to the charity component of the device [48].

#### Intervention Summary

Of the 15 articles, 11 (73%) reported on behavior change, with 3 (23%) relating to the same intervention [37-44,47-49]. Of these, most (n=9, 69%) used the wearables to motivate an increase in PA. The devices were primarily used to provide immediate activity feedback to users, with step count being the most common data point displayed on all devices. Moreover, 2 of the 13 (15%) studies used smartwatches to deliver personalized self-regulation strategies to children with ASD [37,43]. One (8%) study used the wearable to measure and visualize asthma attack risk via a dragon animation [46]. In 2 (15%) of the studies, the wearables were used primarily for research purposes. Wing et al [45] aimed to identify the parameters for determining valid wear time (a measure of how much the device is worn to determine typical patterns of activity) of the smartwatch, and Schaefer et al [35] investigated the feasibility of different wearables to inform future research on measuring PA in children.

The study periods ranged from 4 days to 2 months. Most (9/13, 69%) studies were stand-alone wearable interventions while 4 (31%) studies used the wearables as part of a wider intervention [38,40,46,47]. These included additional behavior change techniques such as motivational and educational messages (n=3, 23%) or required additional equipment as part of the wearable intervention such as a spirometer (n=1, 8%).

#### Smartwatch Software

Using the taxonomy of app features [29], the most common behavior change strategy incorporated into the device was activity feedback (eg, displaying step count), which was reported in 77% (10/13) of the studies. Other features included having preset activity goals and reminders. One study used the built-in SMS text messaging functionality of the Apple Watch to deliver the intervention.

In total, 2 (15%) of the 13 studies used a bespoke or modified software design using an existing commercial smartwatch [43,46]. In one study, a smartwatch system was used to assist with behavior challenges due to emotional dysregulation. The system displays self-regulation activities selected by the children’s caregivers (eg, parents and teachers) via a partner device on the watch face [43]. Hosseini et al [46] described the development of a smartwatch system, which calculates and communicates asthma attack risk. Animated dragon graphics were designed based on previous research, which has shown that animations are a more engaging, age-appropriate design for children [46].

#### Software on Partner App

Overall, 73% (11/15) of the studies (3 refer to the same intervention) described the use of a partner app or web-based dashboard linked to the wearable. Of these, 55% (6/11) reported that the wearable was paired with a parent’s smartphone [40-43,45,46] and 27% (3/11) did not report this information [39,44,49]. For the remaining 18% (2/11) of the studies, children had primary control via a website [36] or were given a compatible tablet [48]. In one study, children were not allowed to use the Fitbit mobile app or web-based dashboard alone (including on their own devices) in line with Fitbit’s privacy policy [45].

The partner apps commonly had more features than the wearable device, such as access to greater data granularity, games, and access rewards. Some of the apps also allowed users to compare their performance against others and set activity goals.

#### Data Sharing Features

Of the 15 studies, 7 (47%; 3 reporting on the same intervention) explicitly reported that the primary data collected via the wearable were shared with other stakeholders [38,40-42,45-47]. These included parents or carers, clinicians, teachers, and peers. Data sharing was a component of the intervention to target behavior change. This included step count being shared in the classroom with peers and teachers [38,47] or among the wider family unit via step-count leaderboards within the partner app to try and encourage increased PA [40]. As part of the wearable system for asthma [46], the authors described an additional interface for physicians to access deidentified data collected via the wearable system, though this was not evaluated. In 3 interventions, parents had control over the wearable’s partner app and thus had access to the data collected via the wearable [40-42,46]. Only 2 (13%) of the 15 articles in this review reported any user feedback relating to data sharing [41,44].

### RQ3: What Is the Feasibility and Acceptability of Using Smartwatch and Activity Trackers for Health-Related Applications with Children Aged 5 to 11 Years?

#### Overview

In total, 3 (20%) of the 15 included studies did not report on the wearable’s acceptability and feasibility or collect any user feedback on this [38,47,48]. 6 of the 15 articles (40%) reported gathering user feedback directly from children [35,36,44-46,49]. Feedback was also provided by parents (n=7, 47%), and in one study, treatment acceptability was completed by staff at the children’s school [37].

It was not possible to statistically synthesize quantitative measures of acceptability and feasibility (response rate and wear time) as there was heterogeneity across the studies in how these statistics were reported. Instead, we provide a descriptive overview.

#### Quantitative Data on Usability, Acceptability, and Feasibility

##### Total Device Wear Time

Of the 13 studies, 5 (38%) [35,36,38,39,45] explicitly reported instructing participants on wear time. This included encouraging participants to wear the wearables as much as possible and specifying a minimum time for wearing or engaging with the partnering app or website. Only 1 (8%) study reported contacting participants if there were no new data for 3 days [45].

In total, 2 (13%) of the 15 studies reported quantitative data on device wear time. In one article, the authors reported the average number of valid recording days (≥1000 steps/d) per week as an indicator of wearable use across the 4-week intervention. This showed that wearable use was generally high, ranging from 71% (wk 3) to 91% (wk 2) [44]. Similarly, in the other article, the authors reported that during the 42-day intervention, the mean number of recording days from the children’s wearables was 36.5 (SD 8.3) days [40].

In total, 2 (13%) of the 15 studies used self-reported data to indicate device use. First, parental logging was used to capture when the device was removed, which authors reported as the children’s level of compliance with the study protocol. The device was reported as being used 98% of the time during the 5 days of data collection [35]. Second, the children’s self-reports indicated that 74% of them checked the device several times per day for updates on their activity. In addition, 62% of children reported removing the device daily and 29% reported forgetting to put it back on [45].

##### Software Use

In total, 2 (13%) of the 15 articles quantitatively reported on partner app use. One study detailed children’s use as indicated by parents in a web-based survey. While the article primarily focused on parents’ use, the authors reported that 42% of children used the Garmin app 2 to 6 times per week during the study [42]. When using the Fitbit Charge HR [45], 80% of children reported using the associated app to see activity information during the study period.

##### Experience Using the Devices

Of the 15 studies, 5 (33%) provided quantitative user feedback on the acceptability and feasibility of using the devices and associated software [35,37,42,44,45], most of which were collected via user surveys or questionnaires. Moreover, 1 (7%) article reported the frequency of common interview responses [35].

After using the Fitbit Charge HR, 98% of children reported feeling comfortable wearing the device among their peers, 87% enjoyed wearing the device over the deployment period, and the majority (98%) would be interested in wearing the device for longer in the future [45]. Most children (23/24, 96%) rated the Movband as the preferred device compared to the wrist-worn tracker without a display, and the hip-worn tracker (not discussed in this review) due to its comfort (10/23, 43%) and its feedback features (8/23, 35%) [35]. Parents reported the perceived usefulness of the Garmin Vivofit JR device and partner app, with 85.9% and 76.6% of parents rating it as very or quite useful, respectively [42]. In the visual directive intervention for ASD by O’Brien et al [37], the overall intervention was evaluated by school staff via a treatment acceptability survey with an average score of 42.75 out of 50. All participants agreed that it was an acceptable method for school staff to deliver directives; however, 2 participants did not think that the treatment was likely to result in permanent improvement.

In total, 2 (13%) of the 15 studies described experiencing data loss due to difficulties with the wearables, such as syncing errors leading to data corruption, user error, or insufficient wear [9,15]. In a study, the authors reported that 1 family withdrew their participation due to issues with setting up the devices [44].

#### Qualitative Data on Usability, Acceptability, and Feasibility

##### Overview

Of the 15 studies, 7 (47%) included qualitative data on users’ experience of using the devices and therefore were included in the thematic synthesis [35,36,39,41-44,49]. Of the 7 studies which collected qualitative data, 5 (71%) provided quotations from participants, which can be found in Table 5. We identified 3 themes: the perceived ease of use (TAM), perceived usefulness (TAM), and the perceptions of engagement.

**Table 5:** Participant quotes extracted from studies (primary data).

Theme, study, and supporting quotes				Participant
**Perceived ease of use**				
	**Schoeppe et al [41], 2023**			
		“Sometimes it wouldn’t sync properly or I thought it was syncing and I am not sure if it actually did”	Parent	
		“My son was getting a bit of a rash from wearing it all the time.. it would come undone really easily”	Parent	
		“I didn’t find it particularly easy but then I am not really technology proficient”	Parent	
	**Creaser et al [44], 2022**			
		“It hurt and it—it made a mark on my arm”	Child aged 5 years	
**Perceived usefulness**				
	**Creaser et al [44], 2022**			
		“I don’t really understand the calories that I’ve burnt. But when I am older I’ll probably understand more”	Child aged 10 years	
		“It really did depend if I was in class or not [if they responded to ‘reminders to move’] because I couldn’t just run out of class to get the steps...sometimes if fit into my break so that would be good”	Child aged 9 years	
		“He’s only 5 if he was a little bit older, I would consider getting him one”	Parent	
		“Maybe when I am older, but I don’t think so right now”	Child aged 10 years	
		“It had like 2 more steps to do so I did those two more steps and then noticed that it wasn’t picking it up”	Child aged 10 years	
		“Well I didn’t really want to compare them because I thought they might be a bit more than me...I was going to do a thing where at the end of the day there’s a winner for how many—for the biggest amount of steps, but I quitted that because I saw mum did a lot of steps at work...it made me jealous”	Child aged 7 years	
	**Masteller et al [36], 2017**			
		“The MovBand I didn’t really like it because you couldn’t realty do anything active, you just look at it which wasn’t really fun to me. It did have one advantage which was seeing if you’re average or not average”	Child aged 9 years	
		“This one I thought was the least interesting because you could barely do anything”	Child aged 10 years	
	**Schoeppe et al [41], 2023**			
		“My son often got told by the teacher to put it away as it was a bit distracting for him in the classroom”	Parent	
		“It actually made us a little more aware of our child’s sleep patterns. Something we’ve investigated a little further”	Parent	
		“Kids were disappointed when swimming and cycling did not record as steps but they could have still entered them”	Parent	
		“Difficulty came when the kids wanted to win...[child] is cheating because he is moving his arm around...but he is going to be top of the leaderboard”	Parent	
	**Saksono et al [39], 2018**			
		“I don’t think hers is accurate. Because sometimes if tells you, ‘she’s at 10,000 steps,’ I’m like ‘you didn’t do 10,000 steps. Like I moved more than you did’”	Parent	
	**Saksono et al [49]** **, 2019**			
		“We just look at them [...] I mean, it’s pretty much like just look at the, see what the numbers say. Um and it’s I leave it”	Parent	
		“I’ve told them, but they be so into their the world. [...] They just be like brushing me off. [...] They’re used to walking around, and moving and activities. [...] They don’t know that they’re completing the things on the challenges [in the Kid Power app]”	Parent	
**Experience using the devices**				
	**Creaser et al [44], 2022**			
		“I already saw by the end of the week the novelty was wearing off”	Parent	
		“I liked uh I liked going on walks because you could check how much steps you’ve done”	Child aged 10 years	
		“I like the firework when you get to 10,000”	Child aged 6 years	
		“I didn’t really like the sleep bit in it because of that...because they [parents] could check when I was sleeping”	Child aged 12 years	
	**Saksono et al [49], 2019**			
		“I give [Kid Power app a] 10 [out of 10] because like I love walking. And like I love, love to walk and it gives you missions. And points, which I really love points”	Child aged 9 years	
	**Schoeppe et al [41], 2023**			
		“The kids absolutely loved the kids app with the little activities and getting coins for things”	Parent	

##### Theme 1: Perceived Ease of Use

In total 2 of the 15 (13%) studies reported that children reported finding wearing the devices uncomfortable and, in some cases, wearing them caused skin irritation [41,44]. In addition, parents described finding the devices and partner apps difficult to use and revealed practical and technical challenges with the devices, such as syncing them and the partner app [41,44]

##### Theme 2: Perceived Usefulness

Most parents and children expressed a positive experience of using wearables overall, with many considering using a device again in the future. However, in a study, there was some discussion regarding the appropriateness of using these devices with younger children. For example, it was highlighted that children cannot respond to the hourly “reminder to move” prompts when they conflict with their school schedule. Children in the study also described challenges with interpreting the data output and visual display of the Fitbit device [44]. Some parents also highlighted some negative consequences of using the devices, such as the devices becoming distracting during school and causing negative emotions or sibling conflict due to competition elements [41,44].

Articles reported that users liked receiving feedback on their activity, with many families describing how this helped increase motivation for engaging in PA [39,41,42,44]. However, children and parents highlighted their concerns regarding the accuracy of the devices to be able to detect and record PA [39,44]. In addition, children were disappointed that not all activities (eg, swimming) could be tracked using some of the devices and thus did not contribute to their overall activity summary [41,44].

In a study, parents described facing challenges when trying to get their child to engage with how the wearable works and to explore the additional features of the companion app. In addition, families reported having limited discussions about the PA data collected via the wearables, including the meaning of the data within the context of their overall health and well-being [49].

##### Theme 3: Perceptions of Engagement

Overall, children enjoyed using the wearables and their partner apps. Step count was frequently reported as a popular feature along with gamified features, such as rewards [35,36,41,44,49]. Devices with these additional features were preferred over those that supported little or no interaction [36]. However, some parents reported that children’s engagement with the devices declined over time due to a novelty effect resulting in a loss of interest [41,44].

Only 1 (7%) of the 15 studies described participant comments in relation to sharing data among participants. Parents liked that the trackers captured additional data (eg, sleep patterns) and described using the partner app to monitor their child often without their involvement [41]. However, there was no exploration of children’s comfort with sharing their data with other stakeholders.

## Discussion

### Principal Findings

This systematic review aimed to answer three RQs by identifying (1) the population characteristics of children targeted in health-related wearable interventions, (2) the characteristics of the devices used, and (3) the feasibility and acceptability of using these devices in wearable interventions for children aged 5 to 11 years. We found that most (10/13, 77%) of the studies focused on PA, with only 23% (3/13) focusing on clinical conditions. The devices were typically used to provide activity feedback to support behavior change, with additional features often available on an accompanying app. Many (10/13, 77%) studies included wider stakeholders (parents, teachers, and peers) and data sharing, either as part of the intervention or implicitly due to the device being linked to a partner app. The quantitative and qualitative user feedback shows that using smartwatches for health interventions is acceptable and feasible with children aged 5 to 11 years, though there were common usability issues (eg, discomfort, technical issues, and short battery life). Overall, several studies included in this review lacked sufficient detail for data extraction, were considered not to have a rigorous data analysis procedure, and thus are considered to be of poor quality (Multimedia Appendix 3 provides full quality assessment). Sample sizes varied from n=1 to n=137 and small sizes limit our ability to generalize their findings, which should be rectified in future research. There was a limited focus on child-centered design, with many studies using commercial adult devices that require parent involvement rather than developing bespoke child-friendly designs. Furthermore, when gathering user feedback, children’s views were often not the focus, with 3 of the 12 (25%) studies not gathering feedback from children but instead focusing on their parents.

### Comparison With Prior Work

All of the studies in this review used existing commercial smartwatches and activity-tracking devices. These provide limited data feedback, often only reporting step count on the device. More detailed information was generally only available through a partner app, which was usually under the control of a parent. This is consistent with previous research, which found that children were frustrated by the limited data feedback available in real time due to it being on the app rather than the device [50]. The devices and their partner apps included a number of behavior change techniques, such as feedback or monitoring and goal setting, both of which are consistent with previous child and adult research [51,52]. The studies also identified usability issues, including discomfort, technical challenges such as syncing issues, and logistical challenges such as poor battery life, which is consistent with previous research [50,53,54].

Despite this, the studies in this review reported high levels of engagement and enjoyment using these devices and their partner apps. In total, 2 (13%) of the 15 studies also reported a novelty effect, indicated by declining use across the study period. This effect has also been observed with adults’ use of wearable devices, where it is common to see initial high engagement levels, followed by abandonment after a few months [55]. One potential way to combat abandonment and support motivation is through the use of gamification, as research has shown that gamified apps have longer use than nongamified apps for PA [56]. Furthermore, while the children were mostly content with wearing the devices, in one of the studies participants described that children were not actively engaging with the data collected via the device. This highlights an important distinction between engagement with the data collection process and the use of or reflection on the data collected. According to the staged-based model of personal informatics by Li et al [57], reflection is an important distinct stage that takes place before action, in which individuals can enact behavior change with their new information. Previous research has shown that the way people reflect on their data can be impacted by both the phase of self-tracking and the feedback they receive such as data visualizations, which help individuals gain insights from their data [58]. This is particularly relevant as children can struggle to understand the data collected and visualized on smartwatches [59], which, in turn, may impact their engagement. For example, research with children aged 10 to 15 years has shown that children can misinterpret biofeedback metrics, including heart rate and calories [60,61]. Therefore, further work is needed to understand how to support children’s sensemaking of personal health data collected via wearables.

To ensure that children understand and can reflect on the data being collected via smartwatches, it is important to consider their needs and preferences by including them and other stakeholders in a user-centered design process [62]. Considering the end users’ experience and including these individuals in the design process has been shown to produce technology, which is more engaging and acceptable to users [63,64]. This is particularly relevant when using children’s wearables in health interventions, as research has shown that children conceptualize health differently from adults. It was found that children extended their understanding of health beyond traditional health measures to include other metrics, such as time spent playing outside [59], highlighting the importance of exploring children’s health-tracking interests. However, many studies in this review did not seem to take a user-centered approach to the design and choice of devices in their studies. For example, a few (3/13, 23%) studies in this review outlined the rationale for their choice of wearable and failed to detail the specific design features of the device or software that supported children to use it in relation to their health. In addition, most of the devices used across the studies were adult devices and not designed specifically for use by children. Moreover, the device partner apps included additional features and behavior change techniques, such as leaderboards, games, and rewards; however, these were often only accessible via a parent’s smartphone as young children did not have their own smartphone. This can lead to the issue identified in previous research of parents acting as “gatekeepers” to their child’s interaction with an intervention. This dynamic can result in children no longer being the focus of the design and generates additional “invisible” work for parents [50,51].

However, including parents in pediatric behavioral interventions has previously been shown to be a key component for achieving sustained behavioral change [8,12]. In this review, 77% (10/13) of the studies included additional stakeholders such as parents and teachers, but it was rare that they considered the child’s perspective on their inclusion. Ethical and privacy issues around collecting and sharing self-tracked data via wearables are rarely discussed [65]. Potapov and Marshall [66] found that teenagers had concerns about the consequences of sharing their data with others; however, this has not been thoroughly investigated in younger children. In this review, privacy concerns were not directly explored or raised by younger children. However, previous research that has investigated the impact of sharing children’s PA and sleep data with their parents or carer found that children reported being uncomfortable with the surveillance. Furthermore, shared access to the data appeared to influence trust between children and their parents or carers negatively, as parents used the data to assert further parental control and impose additional restrictions [67]. Similarly, when using co-design methods with families with children aged 7 to 15 years, it was found that parents and children raised concerns about the impact of data sharing on children’s privacy and autonomy, to which the authors suggested the need for child-controlled data sharing options to help set boundaries and minimize conflict [68]. As these privacy concerns were raised in studies with children older than the focus of this review, this may be indicative of a change that occurs during the transition to adolescence, whereby children are working toward increased independence and autonomy [69]. A robust investigation of end users’ views (children, parents, carers, and other stakeholders) on wearable data sharing and data privacy is an important research gap, with significant implications for design consideration for wearable interventions for children. This should be considered in future research, where it would be valuable to investigate both children’s current preferences for data sharing and privacy as well as their shifting views as they mature and the sociotechnical design considerations that could meet these changing needs.

In this review, most (9/13, 69%) studies focused on using smartwatches and activity trackers to promote PA. Future work should consider the potential of using these devices for more novel health applications. One growing use case for smartwatches within health care and health care research is just-in-time adaptive interventions (JITAIs) [70,71]. JITAIs use mobile and sensing technology to offer personalized and timely support [72] and have been used in a variety of adult and adolescent health domains including PA [70] and substance use behavior [71,73]. In this review, 23% (3/13) of the studies used the devices to deliver interventions in real time. This included using the watch display and SMS text messages to deliver emotional regulation strategies to children with ASD. However, most of the devices in this review only provided basic data summaries on the watch face. Only one study reported the use of a novel visualization using data analytics, in which an animated dragon was used to convey a risk score as part of a JITAI. This highlights the immense potential for novel uses of smartwatches in pediatric health and further work is needed to explore how smartwatch interventions can be adapted to be developmentally appropriate to convey health information to children in a way that is meaningful to support behavior change.

### Strengths and Limitations

Digital health is an interdisciplinary field, and a key strength of this review is the interdisciplinary approach taken by searching both health science databases as well as human-computer interaction venues. The review aimed to investigate how wearables are designed and used for children (5-11 y) to understand developmentally sensitive design implications. To investigate this, we looked across the broad spectrum of child health behaviors and conditions, which means our review is not limited to PA interventions, making the findings relevant to a broader scope of researchers working within pediatric wearable interventions. However, we excluded studies that included our target age group (5-11 y) but also included older and younger children (eg, 5-18 y) that did not report the results separately, as it was not feasible to stratify the results by age. We also conducted a narrative synthesis, which has previously been criticized for lacking transparency [74]. To mitigate this, we have included primary data where possible in our qualitative summary. The limited qualitative data available in the articles also resulted in our synthesized themes having limited richness.

### Conclusions

This systematic review addresses the gap in understanding children’s wearables by expanding the scope of the review to a broad range of health applications, with a focus on identifying developmentally sensitive design features and understanding the experience of younger children. We identified a total of 15 articles (13 distinct studies) that involved children aged 5 to 11 years using a smartwatch for health-related purposes. Most of these were using commercial smartwatches and activity trackers as tools to help increase PA, but other novel applications included uses to support children with ASD and asthma. Of those that gathered user feedback, experiences using the devices were positive, although there was an indication that the children may not have been engaging with the visualization of their data. Overall, the studies lacked a child-centered focus with (1) many (9/15, 60%) studies not including children’s opinions reflecting on the designs, (2) most of the functionality and data visualization was placed on partner apps usually under the control of parents, and (3) most smartwatches and activity trackers being used were adult devices. Further work is needed to better understand children’s perspectives on the design and evaluation of these devices while also investigating other potential areas of research beyond PA tracking for children, such as managing chronic health conditions. In addition, future research should provide richer details about the devices used, including the rationale for the choice of device, and who has access to the data to consider how this aligns with their research aim and user experience.

## Appendix

Multimedia Appendix 1 PRISMA (Preferred Reporting Items for Systematic Reviews and Meta-Analyses) checklist.

Multimedia Appendix 2 Search strategy.

Multimedia Appendix 3 Taxonomy of app features (as reported).

Multimedia Appendix 4 Quality assessment.

## Abbreviations

ASD: autism spectrum disorder
JITAI: just in-time adaptive intervention
PA: physical activity
PRISMA: Preferred Reporting Items for Systematic Reviews and Meta-Analyses
RQ: research question
TAM: technology acceptance model

## References

[1] Kessler RC, McLaughlin KA, Green JG, Gruber MJ, Sampson NA, Zaslavsky AM, Aguilar-Gaxiola S, Alhamzawi AO, Alonso J, Angermeyer M, Benjet C, Bromet E, Chatterji S, de Girolamo G, Demyttenaere K, Fayyad J, Florescu S, Gal G, Gureje O, Haro JM, Hu C, Karam EG, Kawakami N, Lee S, Lépine JP, Ormel J, Posada-Villa J, Sagar R, Tsang A, Ustün TB, Vassilev S, Viana MC, Williams DR (2010). Childhood adversities and adult psychopathology in the WHO World Mental Health Surveys. Br J Psychiatry.

[2] Wadsworth ME, Kuh DJ (1997). Childhood influences on adult health: a review of recent work from the British 1946 national birth cohort study, the MRC National Survey of Health and Development. Paediatr Perinat Epidemiol.

[3] Galobardes B, Smith GD, Lynch JW (2006). Systematic review of the influence of childhood socioeconomic circumstances on risk for cardiovascular disease in adulthood. Annals of Epidemiology.

[4] Nader PR, O'Brien M, Houts R, Bradley R, Belsky J, Crosnoe R, Friedman S, Mei Z, Susman EJ, National Institute of Child Health and Human Development Early Child Care Research Network (2006). Identifying risk for obesity in early childhood. Pediatrics.

[5] Dalton ED, Hammen CL, Brennan PA, Najman JM (2016). Pathways maintaining physical health problems from childhood to young adulthood: the role of stress and mood. Psychol Health.

[6] Delaney L, Smith JP (2012). Childhood health: trends and consequences over the life course. Future Child.

[7] Van Cleave J, Gortmaker SL, Perrin JM (2010). Dynamics of obesity and chronic health conditions among children and youth. JAMA.

[8] Brigden A, Parslow RM, Linney C, Higson-Sweeney N, Read R, Loades M, Davies A, Stoll S, Beasant L, Morris R, Ye S, Crawley E (2019). How are behavioural interventions delivered to children (5-11 years old): a systematic mapping review. BMJ Paediatr Open.

[9] Rose T, Barker M, Maria Jacob C, Morrison L, Lawrence W, Strömmer S, Vogel C, Woods-Townsend K, Farrell D, Inskip H, Baird J (2017). A systematic review of digital interventions for improving the diet and physical activity behaviors of adolescents. J Adolesc Health.

[10] Wang J, Wang Y, Wei C, Yao N, Yuan A, Shan Y, Yuan C (2014). Smartphone interventions for long-term health management of chronic diseases: an integrative review. Telemed J E Health.

[11] McDermott MS, While AE (2013). Maximizing the healthcare environment: a systematic review exploring the potential of computer technology to promote self-management of chronic illness in healthcare settings. Patient Educ Couns.

[12] Brigden A, Anderson E, Linney C, Morris R, Parslow R, Serafimova T, Smith L, Briggs E, Loades M, Crawley E (2020). Digital behavior change interventions for younger children with chronic health conditions: systematic review. J Med Internet Res.

[13] Piwek L, Ellis DA, Andrews S, Joinson A (2016). The rise of consumer health wearables: promises and barriers. PLoS Med.

[14] Butler S, Sculley D, Santos D, Girones X, Singh-Grewal D, Coda A (2024). Using digital health technologies to monitor pain, medication adherence and physical activity in young people with juvenile idiopathic arthritis: a feasibility study. Healthcare (Basel).

[15] Zehrung R, Huang L, Lee B, Choe EK Investigating opportunities to support kids' agency and well-being: a review of kids' wearables. arXiv.

[16] Radesky JS, Weeks HM, Ball R, Schaller A, Yeo S, Durnez J, Tamayo-Rios M, Epstein M, Kirkorian H, Coyne S, Barr R (2020). Young children's use of smartphones and tablets. Pediatrics.

[17] Reeder B, David A (2016). Health at hand: a systematic review of smart watch uses for health and wellness. J Biomed Inform.

[18] Jat AS, Grønli TM (2022). Smart watch for smart health monitoring: a literature review. Proceedings of the 9th International Work-Conference on Bioinformatics and Biomedical Engineering.

[19] Grave J, Blissett J (2004). Is cognitive behavior therapy developmentally appropriate for young children? A critical review of the evidence. Clin Psychol Rev.

[20] Erickson SJ, Gerstle M, Feldstein SW (2005). Brief interventions and motivational interviewing with children, adolescents, and their parents in pediatric health care settings: a review. Arch Pediatr Adolesc Med.

[21] Yardley L, Morrison L, Bradbury K, Muller I (2015). The person-based approach to intervention development: application to digital health-related behavior change interventions. J Med Internet Res.

[22] Gram-Hansen SB (2019). Family wearables – what makes them persuasive?. Behav Inf Technol.

[23] Liberati A, Altman DG, Tetzlaff J, Mulrow C, Gøtzsche PC, Ioannidis JP, Clarke M, Devereaux PJ, Kleijnen J, Moher D (2009). The PRISMA statement for reporting systematic reviews and meta-analyses of studies that evaluate health care interventions: explanation and elaboration. Ann Intern Med.

[24] Smith PG, Morrow RH, Ross DA (2015). Field Trials of Health Interventions: A Toolbox.

[25] Henriksen A, Haugen Mikalsen M, Woldaregay AZ, Muzny M, Hartvigsen G, Hopstock LA, Grimsgaard S (2018). Using fitness trackers and smartwatches to measure physical activity in research: analysis of consumer wrist-worn wearables. J Med Internet Res.

[26] Popay J, Roberts H, Sowden A, Petticrew M (2006). Guidance on the conduct of narrative synthesis in systematic reviews: a product from the ESRC methods programme. Lancaster University.

[27] Noyes J, Booth A, Cargo M, Flemming K, Harden A, Harris J, Garside R, Hannes K, Pantoja T, Thomas J, Higgins JP, Thomas J, Chandler J, Cumpston M, Li T, Page MJ, Welch VA (2019). Qualitative evidence. Cochrane Handbook for Systematic Reviews of Interventions.

[28] Davis FD (1993). User acceptance of information technology: system characteristics, user perceptions and behavioral impacts. Int J Man Mach Stud.

[29] Villalobos-Zúñiga G, Cherubini M (2020). Apps that motivate: a taxonomy of app features based on self-determination theory. Int J Hum Comput Stud.

[30] Jansen-Kosterink S, Broekhuis M, van Velsen L (2022). Time to act mature-Gearing eHealth evaluations towards technology readiness levels. Digit Health.

[31] C.A.S.P. qualitative studies checklist. Critical Appraisal Skills Programme (CASP).

[32] Protogerou C, Hagger MS (2020). A checklist to assess the quality of survey studies in psychology. Methods Psychol.

[33] Quality assessment of controlled intervention studies. National Heart, Lung, and Blood Institute.

[34] Quality assessment tool for before-after (pre-post) studies with no control group. National Heart, Lung, and Blood Institute.

[35] Schaefer SE, Van Loan M, German JB (2014). A feasibility study of wearable activity monitors for pre-adolescent school-age children. Prev Chronic Dis.

[36] Masteller B, Sirard J, Freedson P (2017). The physical activity tracker testing in youth (P.A.T.T.Y.) study: content analysis and children's perceptions. JMIR Mhealth Uhealth.

[37] O'Brien AM, Schlosser RW, Shane H, Wendt O, Yu C, Allen AA, Cullen J, Benz A, O'Neill L (2020). Providing visual directives via a smart watch to a student with autism spectrum disorder: an intervention note. Augment Altern Commun.

[38] Buchele Harris H, Chen W (2018). Technology-enhanced classroom activity breaks impacting children's physical activity and fitness. J Clin Med.

[39] Saksono H, Castaneda-Sceppa C, Hoffman J, El-Nasr MS, Morris V, Parker AG (2018). Family health promotion in low-SES neighborhoods: a two-month study of wearable activity tracking. Proceedings of the 2018 CHI Conference on Human Factors in Computing Systems.

[40] Schoeppe S, Salmon J, Williams SL, Power D, Alley S, Rebar AL, Hayman M, Duncan MJ, Vandelanotte C (2020). Effects of an activity tracker and app intervention to increase physical activity in whole families-the step it up family feasibility study. Int J Environ Res Public Health.

[41] Schoeppe S, Waters K, Salmon J, Williams SL, Power D, Alley S, Rebar AL, Hayman M, Duncan MJ, Vandelanotte C (2023). Experience and satisfaction with a family-based physical activity intervention using activity trackers and apps: a qualitative study. Int J Environ Res Public Health.

[42] Schoeppe S, Salmon J, Williams S, Power D, Waters K, Alley S, Rebar AL, Hayman M, Duncan MJ, Vandelanotte C (2022). Feasibility of using activity trackers and apps to increase physical activity in whole families: the step it up family intervention. Digit Health.

[43] Torrado JC, Gomez J, Montoro G (2017). Emotional self-regulation of individuals with autism spectrum disorders: smartwatches for monitoring and interaction. Sensors (Basel).

[44] Creaser AV, Hall J, Costa S, Bingham DD, Clemes SA (2022). Exploring families' acceptance of wearable activity trackers: a mixed-methods study. Int J Environ Res Public Health.

[45] Wing D, Godino JG, Baker FC, Yang R, Chevance G, Thompson WK, Reuter C, Bartsch H, Wilbur A, Straub LK, Castro N, Higgins M, Colrain IM, de Zambotti M, Wade NE, Lisdahl KM, Squeglia LM, Ortigara J, Fuemmeler B, Patrick K, Mason MJ, Tapert SF, Bagot KS (2022). Recommendations for identifying valid wear for consumer-level wrist-worn activity trackers and acceptability of extended device deployment in children. Sensors (Basel).

[46] Hosseini A, Buonocore C, Hashemzadeh S, Hojaiji H, Kalantarian H, Sideris C, Bui A, King C, Sarrafzadeh M (2017). Feasibility of a secure wireless sensing smartwatch application for the self-management of pediatric asthma. Sensors (Basel).

[47] Jackson SL, Kraft GL, Khoo M, Rockie P, Karin C (2022). Enhancing the fitness and academics of children using technology in the schools (FACTS). J Phys Educ Sport.

[48] Duck AA, Hall KC, Klamm M, Temple M, Robinson JC (2021). Physical activity and fitness: the feasibility and preliminary effectiveness of wearable activity tracker technology incorporating altruistic motivation in youth. J Spec Pediatr Nurs.

[49] Saksono H, Castaneda-sceppa C, Hoffman J, El-Nasr MS, Morris V, Parker A (2019). Social reflections on fitness tracking data: a study with families in low-SES neighborhoods. Proceedings of the 2019 CHI Conference on Human Factors in Computing Systems.

[50] Mackintosh KA, Chappel SE, Salmon J, Timperio A, Ball K, Brown H, Macfarlane S, Ridgers ND (2019). Parental perspectives of a wearable activity tracker for children younger than 13 years: acceptability and usability study. JMIR Mhealth Uhealth.

[51] Oygür I, Epstein DA, Chen Y (2020). Raising the responsible child: collaborative work in the use of activity trackers for children. Proc ACM Hum Comput Interact.

[52] Düking P, Tafler M, Wallmann-Sperlich B, Sperlich B, Kleih S (2020). Behavior change techniques in wrist-worn wearables to promote physical activity: content analysis. JMIR Mhealth Uhealth.

[53] Ridgers ND, McNarry MA, Mackintosh KA (2016). Feasibility and effectiveness of using wearable activity trackers in youth: a systematic review. JMIR Mhealth Uhealth.

[54] Fraser H, Thompson L, Crawley E, Ridd MJ, Brigden A (2024). "not one size fits all": the challenges of measuring paediatric health-related quality of life and the potential role of digital ecological momentary assessment: a qualitative study. Qual Life Res.

[55] Attig C, Franke T (2020). Abandonment of personal quantification: a review and empirical study investigating reasons for wearable activity tracking attrition. Comput Human Behav.

[56] Maher CA, Olds T, Vandelanotte C, Plotnikoff R, Edney SM, Ryan JC, DeSmet A, Curtis RG (2022). Gamification in a physical activity app: what gamification features are being used, by whom, and does it make a difference?. Games Health J.

[57] Li I, Day A, Forlizzi J (2010). A stage-based model of personal informatics systems. Proceedings of the 2010 SIGCHI Conference on Human Factors in Computing Systems.

[58] Choe EK, Lee B, Zhu H, Riche NH, Baur D (2017). Understanding self-reflection: how people reflect on personal data through visual data exploration. Proceedings of the 11th EAI International Conference on Pervasive Computing Technologies for Healthca.

[59] Ananthanarayan S, Siek K, Eisenberg M (2016). A craft approach to health awareness in children. Proceedings of the 2016 ACM Conference on Designing Interactive Systems.

[60] Ankrah EA, Cibrian FL, Silva LM, Tavakoulnia A, Beltran JA, Schuck SE, Lakes KD, Hayes GR (2023). Me, my health, and my watch: how children with ADHD understand smartwatch health data. ACM Trans Comput Hum Interact.

[61] Cibrian FL, Lakes KD, Tavakoulnia A, Guzman K, Schuck S, Hayes GR (2020). Supporting self-regulation of children with ADHD using wearables: tensions and design challenges. Proceedings of the 2020 CHI Conference on Human Factors in Computing Systems.

[62] Wiljén A, Chaplin JE, Crine V, Jobe W, Johnson E, Karlsson K, Lindroth T, Schwarz A, Stenmarker M, Thunberg G, Öhlén J, Nilsson S (2022). The development of an mHealth tool for children with long-term illness to enable person-centered communication: user-centered design approach. JMIR Pediatr Parent.

[63] McCurdie T, Taneva S, Casselman M, Yeung M, McDaniel C, Ho W, Cafazzo J (2012). mHealth consumer apps: the case for user-centered design. Biomed Instrum Technol.

[64] Birnie KA, Campbell F, Nguyen C, Lalloo C, Tsimicalis A, Matava C, Cafazzo J, Stinson J (2019). iCanCope PostOp: user-centered design of a smartphone-based app for self-management of postoperative pain in children and adolescents. JMIR Form Res.

[65] Epstein DA, Caldeira C, Figueiredo MC, Lu X, Silva LM, Williams L, Lee JH, Li Q, Ahuja S, Chen Q, Dowlatyari P, Hilby C, Sultana S, Eikey EV, Chen Y (2020). Mapping and taking stock of the personal informatics literature. Proc ACM Interact Mob Wearable Ubiquitous Technol.

[66] Potapov K, Marshall P (2020). LifeMosaic: co-design of a personal informatics tool for youth. Proceedings of the 2020 Interaction Design and Children Conference.

[67] Jørgensen MS, Nissen FK, Paay J, Kjeldskov J, Skov MB (2016). Monitoring children's physical activity and sleep: a study of surveillance and information disclosure. Proceedings of the 28th Australian Conference on Computer-Human Interaction.

[68] Oygur Ilhan I, Chen Y, Epstein DA (2023). Co-designing for the co-use of child-owned wearables. Proceedings of the 22nd Annual ACM Interaction Design and Children Conference.

[69] Daddis C (2011). Desire for increased autonomy and adolescents' perceptions of peer autonomy: "everyone else can; why can't I?". Child Dev.

[70] Hardeman W, Houghton J, Lane K, Jones A, Naughton F (2019). A systematic review of just-in-time adaptive interventions (JITAIs) to promote physical activity. Int J Behav Nutr Phys Act.

[71] Yang MJ, Sutton SK, Hernandez LM, Jones SR, Wetter DW, Kumar S, Vinci C (2023). A just-in-time adaptive intervention (JITAI) for smoking cessation: feasibility and acceptability findings. Addict Behav.

[72] Nahum-Shani I, Smith SN, Spring BJ, Collins LM, Witkiewitz K, Tewari A, Murphy SA (2018). Just-in-time adaptive interventions (JITAIs) in mobile health: key components and design principles for ongoing health behavior support. Ann Behav Med.

[73] Coughlin LN, Nahum-Shani I, Philyaw-Kotov ML, Bonar EE, Rabbi M, Klasnja P, Murphy S, Walton MA (2021). Developing an adaptive mobile intervention to address risky substance use among adolescents and emerging adults: usability study. JMIR Mhealth Uhealth.

[74] Campbell M, Katikireddi SV, Sowden A, McKenzie JE, Thomson H (2018). Improving conduct and reporting of narrative synthesis of quantitative data (ICONS-Quant): protocol for a mixed methods study to develop a reporting guideline. BMJ Open.

