# Correction: Exploring the Use of Smartwatches and Activity Trackers for Health-Related Purposes for Children Aged 5 to 11 years: Systematic Review

**DOI:** 10.2196/82479

**Published:** 2025-08-27

**Authors:** Lauren Thompson, Sydney Charitos, Jon Bird, Paul Marshall, Amberly Brigden

**Affiliations:** 1 University of Bristol Bristol United Kingdom

In “Exploring the Use of Smartwatches and Activity Trackers for Health-Related Purposes for Children Aged 5 to 11 years: Systematic Review” (J Med Internet Res 2025;27:e62944) one error was noted.

In the published article, the grant number was omitted from the funding information provided in the *Acknowledgements* section. The grant number has now been included, and the *Acknowledgements* section now reads as follows:

This work was supported by the EPSRC Digital Health and Care Centre for Doctoral Training (UKRI Grant No. EP/S023704/1).

The correction will appear in the online version of the paper on the JMIR Publications website, together with the publication of this correction notice. Because this was made after submission to PubMed, PubMed Central, and other full-text repositories, the corrected article has also been resubmitted to those repositories.

