# Supplementary material for: Exploring the Use of Smartwatches and Activity Trackers for Health-Related Purposes for Children Aged 5 to 11 years: Systematic Review
Source: J Med Internet Res. 2025 Jan 27;27:e62944. doi: 10.2196/62944 (PMC11811667; doi:10.2196/62944)
Supplement: Multimedia Appendix 2 [file jmir_v27i1e62944_app2.docx]

Appendix 2: Search Strategy for Embase

1. (Child* or Paediatric* or Pediatric* or Juvenil* or "Minor" or "Minors" or "school child" or "school children" or Schoolchild* or "Kid" or "kids" or Pre-adolescen* or Preadolescen* or "Primary school child" or "primary school children" or "elementary school child" or "elementary school children" or "middle school child" or "middle school children" or "junior school child" or "junior school children" or Pre-teen* or preteen* or pubescen* or pre-pubescen* or prepubescen* or "boy" or "boys" or "girl" or "girls" or stepchild* or step-child* or (young* adj3 child*)).ti,ab,kf.

2.

("wearable electronic devices" or "smartwatch" or "smart watch" or "wearable technology" or "activity tracker" or "fitness tracker" or "wrist wearable" or "wrist-worn wearable" or "wrist watch").ti,ab,kf.

3. 1 AND 2
