# Supplementary material for: Exploring the Use of Smartwatches and Activity Trackers for Health-Related Purposes for Children Aged 5 to 11 years: Systematic Review
Source: J Med Internet Res. 2025 Jan 27;27:e62944. doi: 10.2196/62944 (PMC11811667; doi:10.2196/62944)
Supplement: Multimedia Appendix 3 [file jmir_v27i1e62944_app3.docx]

Taxonomy of App Features (as reported) [1]

| Study | Autonomy-supportive features | | | | Competence supportive features | | | | Relatedness supportive features | | | |
| --- | --- | --- | --- | --- | --- | --- | --- | --- | --- | --- | --- | --- |
|  | Reminders | Motivational messages | Goal setting | Precommitments | Activity feedback | History | Self-monitoring | Rewards | Performance sharing | Peer comparison | Challenge peer | Messaging |
|  | | | | | | | | | | | | |
| O’Brien et al [2] | No | No | No | No | No | No | No | No | No | No | No | Yes^a^ |
| Buchele Harris and Chen [3] | No | No | No | No | Yes^a^ | No | No | No | No | No | No | No |
| Saksono et al [4] | NR^b^ | NR | NR | NR | NR | NR | NR | NR | NR | NR | NR | NR |
| Schoeppe et al [5] | No | No | Yes^c^ | Yes^c^ | Yes^a^ | No | No | Yes^c^ | No | Yes^c^ | No | No |
| Torrado et al [6] | No | Yes^a^ | No | No | Yes^a^ | No | No | No | No | No | No | No |
| Creaser et al [7] | Yes^a^ | No | Yes^a^ | Yes^a^ | Yes^a^ | Yes^c^ | Yes^c^ | Yes^c^ | Yes^c^ | No | No | No |
| Masteller et al [8] | No | No | Yes^c^ | No | Yes^a^ | No | No | Yes^c^ | No | Yes^c^ | No | No |
| Wing et al [9] | NR | NR | NR | NR | NR | NR | NR | NR | NR | NR | NR | NR |
| Hosseini et al [10] | No | No | No | No | Yes^a^ | Yes^c^ | No | No | No | No | No | No |
| Schoeppe et al [11] | No | No | Yes^c^ | Yes^a^ | Yes^a^ | Yes^c^ | Yes^c^ | Yes^c^ | No | Yes^c^ | Yes^c^ | No |
| Jackson et al [12] | NR | NR | NR | NR | NR | NR | NR | NR | NR | NR | NR | NR |
| Schoeppe et al [13] | No | No | No | Yes^a^ | Yes^a^ | No | No | Yes^c^ | No | Yes^c^ | Yes^c^ | No |
| Schaefer et al [14] | No | No | No | No | Yes^a^ | No | No | No | No | No | No | No |
| Duck et al [15] | No | No | No | No | Yes^a^ | No | No | Yes^a^ | No | No | No | No |
| Saksono et al [16] | NR | NR | NR | NR | NR | NR | NR | NR | NR | NR | NR | NR |

^a^Reported to be present in the device.

^b^NR: not reported.

^c^Reported to be present in the partner app.

**References**

1. O’Brien, A.M., et al., *Providing visual directives via a smart watch to a student with Autism Spectrum Disorder: an intervention note.* Augmentative and Alternative Communication, 2020. **36**(4): p. 249-257.
2. Buchele Harris, H. and W. Chen, *Technology-enhanced classroom activity breaks impacting children’s physical activity and fitness.* Journal of clinical medicine, 2018. **7**(7): p. 165.
3. Saksono, H., et al. *Family health promotion in low-SES neighborhoods: A two-month study of wearable activity tracking*. in *Proceedings of the 2018 chi conference on human factors in computing systems*. 2018.
4. Schoeppe, S., et al., *Effects of an activity tracker and app intervention to increase physical activity in whole families—the step it up family feasibility study.* International Journal of Environmental Research and Public Health, 2020. **17**(20): p. 7655.
5. Torrado, J.C., J. Gomez, and G. Montoro, *Emotional self-regulation of individuals with autism spectrum disorders: smartwatches for monitoring and interaction.* Sensors, 2017. **17**(6): p. 1359.
6. Creaser, A.V., et al., *Exploring families’ acceptance of wearable activity trackers: A mixed-methods study.* International Journal of Environmental Research and Public Health, 2022. **19**(6): p. 3472.
7. Masteller, B., J. Sirard, and P. Freedson, *The physical activity tracker testing in youth (PATTY) study: Content analysis and children’s perceptions.* JMIR mHealth and uHealth, 2017. **5**(4): p. e6347.
8. Wing, D., et al., *Recommendations for identifying valid wear for consumer-level wrist-worn activity trackers and acceptability of extended device deployment in children.* Sensors, 2022. **22**(23): p. 9189. 46.
9. Hosseini, A., et al., *Feasibility of a secure wireless sensing smartwatch application for the self-management of pediatric asthma.* Sensors, 2017. **17**(8): p. 1780.
10. Schoeppe, S., et al., *Experience and Satisfaction with a Family-Based Physical Activity Intervention Using Activity Trackers and Apps: A Qualitative Study.* International Journal of Environmental Research and Public Health, 2023. **20**(4): p. 3327.
11. Jackson, S.L., et al., *Enhancing the Fitness and Academics of Children using Technology in the Schools (FACTS).* Journal of Physical Education and Sport, 2022. **22**(11): p. 2810-2817.
12. Schoeppe, S., et al., *Feasibility of using activity trackers and apps to increase physical activity in whole families: The Step it Up Family intervention.* Digital Health, 2022. **8**: p. 20552076221129083.
13. Schaefer, S.E., M. Van Loan, and J.B. German, *Peer reviewed: A feasibility study of wearable activity monitors for pre-adolescent school-age children.* Preventing chronic disease, 2014. **11**.
14. Duck, A.A., et al., *Physical activity and fitness: the feasibility and preliminary effectiveness of wearable activity tracker technology incorporating altruistic motivation in youth.* Journal for Specialists in Pediatric Nursing, 2021. **26**(1): p. e12313.
15. Saksono, H., et al. *Social reflections on fitness tracking data: A study with families in low-SES neighborhoods*. in *Proceedings of the 2019 chi conference on human factors in computing systems*. 2019.
